# Treatment of Dairy Wastewater Retentate After Microfiltration: Evaluation of the Performance of the System Based on Activated Sludge and Activated Carbon

**DOI:** 10.3390/membranes15080237

**Published:** 2025-08-06

**Authors:** Maciej Życki, Wioletta Barszcz, Monika Łożyńska

**Affiliations:** 1 Łukasiewicz Research Network—Institute for Sustainable Technologies, Pułaskiego 6/10, 26-600 Radom, Poland; maciej.zycki@itee.lukasiewicz.gov.pl (M.Ż.); monika.lozynska@itee.lukasiewicz.gov.pl (M.Ł.); 2 Textile Institute, Faculty of Material Technologies and Textile Design, Lodz University of Technology, Żeromskiego St. 116, 90-924 Lodz, Poland; 3 Faculty of Environmental Engineering, Warsaw University of Technology, Nowowiejska 20, 00-653 Warszawa, Poland

**Keywords:** dairy wastewater, microfiltration, activated sludge, activated carbon, hybrid treatment, nutrient removal

## Abstract

The dairy industry generates significant amounts of wastewater, including microfiltration (MF) retentate, a byproduct thickened with organic and inorganic pollutants. This study focuses on the treatment of two times concentrated MF retentate using a hybrid system based on biological treatment in a sequential batch reactor (SBR) and adsorption on activated carbon. The first stage involved cross-flow microfiltration using a 0.2 µm PVDF membrane at 0.5 bar, resulting in reductions of 99% in turbidity and 79% in chemical oxygen demand (COD), as well as a partial reduction in conductivity. The second stage involved 24-h biological treatment in a sequential batch reactor (SBR) with activated sludge (activated sludge index: 80 cm^3^/g, MLSS 2500 mg/dm^3^), resulting in further reductions in COD (62%) and TOC (30%), as well as the removal of 46% of total phosphorus (TP) and 35% of total nitrogen (TN). In the third stage, the decantate underwent adsorption in a column containing powdered activated carbon (PAC; 1 g; S_(BET) = 969 m^2^ g^−1^), reducing the concentrations of key indicators to the following levels: COD 84%, TOC 70%, TN 77%, TP 87% and suspended solids 97%. Total pollutant retention ranged from 24.6% to 97.0%. These results confirm that the MF–SBR–PAC system is an effective, compact solution that significantly reduces the load of organic and biogenic pollutants in MF retentates, paving the way for their reuse or safe discharge into the environment.

## 1. Introduction

The food industry is one of the fastest-growing sectors of Poland’s national economy. It is primarily focused on animal production, including dairy products, which account for around 7% of all agricultural products intended for export. In 2022, Poland’s cow’s milk production amounted to nearly 15 billion dm^3^ [1]. In addition to its large processing capacity, the dairy industry is characterised by significant water consumption. Producing 1 dm^3^ of milk uses between 0.2 and 20 dm^3^ of water, depending on the enterprise’s size and the manufactured end product [2]. The dairy industry generates waste (both solid and liquid) exceeding the volume of milk produced by a factor of three. It is estimated that milk processing generates almost 4 million cubic metres of wastewater and over 11 million cubic metres of solid waste each year [3,4]. In Europe, the dairy industry, in particular, is considered the largest source of industrial wastewater. In 2019, the EU produced 167.4 million tonnes of milk, with wastewater amounting to 192.5 million m^3^, and the estimated value of sludge was approximately 2 kg for every 0.1 m^3^ of milk [3,5,6,7,8,9].

Most of the wastewater generated in dairies originates from production processes (accounting for 50–80% of total water consumption). The production of certain types of dairy products requires separate technological lines. Consequently, the composition of dairy wastewater varies, differing in both quantity and quality, which significantly hinders the operation of on-site wastewater treatment plants [6,10]. Additionally, the daily volume of dairy wastewater can vary significantly due to the periodic cleaning and rinsing of production lines [11,12]. In addition to cleaning and sanitising agents, dairy wastewater primarily contains organic pollutants, including milk, lactose, whey, and fat residues [13,14,15]. Such wastewater undergoes rapid fermentation and biodegradation, posing an environmental threat due to the release of large amounts of H_2_S and ammonia, as well as the potential for uncontrolled microbial growth [10].

Given this, treating wastewater from the dairy industry is of key importance due to its environmental impact. Various treatment methods are currently used, including biological, physical and chemical processes.

Biological methods based on activated sludge are among the most commonly used techniques for treating dairy wastewater. These aerobic processes enhance the activity of microorganisms responsible for decomposing organic matter, resulting in a decrease in indicators, such as biochemical oxygen demand (BOD) and chemical oxygen demand (COD). Studies indicate that activated sludge systems are highly effective at removing organic pollutants, although results may vary. For example, in the study presented by Kaewsuk et al. (2010) [16], where the kinetic coefficients of mixed cultures of purple photosynthetic bacteria using dairy wastewater in MSBR were verified, an 81% COD reduction was achieved [16]. In the study by Sivrioğlu and Yonar (2015) [17], who analysed the toxicity of pre-treated dairy wastewater using the Fenton method on activated sludge microorganisms, a BOD reduction of 99% was achieved [17].

Despite the numerous advantages of using activated sludge, potential operational problems, such as sludge bulking, high production of secondary sludge, sensitivity to changes in wastewater load and quality, high energy consumption, and problems with the removal of specific compounds, must be considered, as these can interfere with the treatment process. Measures to mitigate the negative effects of sludge bulking include optimising aeration conditions [17].

Recent studies show that combining anaerobic and aerobic processes can increase treatment efficiency while reducing energy consumption. This approach combines the advantages of both systems, providing an effective solution to the problem of variable wastewater composition, which is characteristic of the dairy industry [18,19].

Dairy wastewater treatment can be carried out using various technologies. Membrane techniques are one of the modern methods of treating this type of wastewater. Membrane techniques are also utilised in the dairy industry for technological processes, such as the production of whey protein [20]. Membrane filtration, including processes such as microfiltration (MF), ultrafiltration (UF) and reverse osmosis (RO), is becoming increasingly popular for treating dairy wastewater due to its high efficiency in removing a wide range of contaminants, including fats, proteins and dissolved chemicals [21,22].

These processes not only enable treatment but also recover valuable components from the dairy wastewater stream. Combining membrane technologies with biological systems (e.g., membrane bioreactors (MBRs)) enables the effective removal of pollutants and optimal management of biomass retention [23].

The primary challenge when employing membrane techniques to treat wastewater from the dairy industry is fouling, which necessitates further research into solutions, such as turbulence promoters or membrane surface modifications, to mitigate contamination [24,25].

Another physical method for treating dairy wastewater is the use of adsorption-based processes. These methods are particularly useful for removing organic compounds, fats, and phosphates. The advantages of adsorption methods in treating dairy wastewater include their technological simplicity, the ability to regenerate some sorbents, and rapid treatment results even with variable wastewater composition. In a study by Mishra et al. (2024) [26], activated coconut shell carbon was used to treat synthetic dairy wastewater. A 76% COD removal and 82% BOD removal were achieved at a concentration of 150 mg/dm^3^ and a contact time of 120 min [26].

The integration of biotechnological methods with membrane technologies presents promising opportunities for enhancing the efficiency of wastewater treatment. Current research focuses, among other things, on the synergistic use of advanced oxidation processes and biological methods [27], which enables the maximisation of organic matter removal while minimising energy and operating costs [21]. The implementation of hybrid membrane systems shows potential for further improvement in treatment efficiency, particularly in the context of nutrient recovery [28,29,30].

The study aimed to assess the possibility of utilising retentates produced after the microfiltration of dairy wastewater from the cleaning of industrial installations using combined treatment methods: activated sludge and sorption on an activated carbon bed. In the experimental work, the SBR principle was used for biological treatment, which involves a gradual change in process conditions. 

## 2. Materials and Methods

### 2.1. Materials

The tests were conducted on typical dairy wastewater originating from the cleaning of a production line using lye and sodium hypochlorite, which processes milk and other dairy products. The selected dairy wastewater comes from a production plant located in Poland, in the Mazowieckie Province. Table 1 presents the physicochemical characteristics of the wastewater.

The MF process was performed using spiral-wound membranes designed for cross-flow processes (Table 2).

The activated sludge used in the biological treatment process also came from the local production plant from which dairy wastewater was collected. The volume index of the sludge necessary to determine its activity was determined according to the accepted formula:

(1) $$I=\;\frac{V_{OS}}{V\times \;S_{s}}\;,$$

where:

I—activated sludge index, (cm^3^ × g^−1^);
V_OS_—sediment volume after 30 min of thickening (cm^3^);
S_S_—activated sludge concentration in the cylinder, (g_sm_/cm^3^);
V—initial sludge volume (cm^3^).

The decantates obtained after biological purification were subjected to adsorption on powdered activated carbon (PAC; VWR). The selected adsorbent material had a particle size of 50–75 µm and was produced through high-temperature pyrolysis of a mixture of vegetable and wood waste. The specific surface area, presence of functional groups and structural order were determined in this study. As part of the research, the basic structural properties of the sorbent material used were characterised. 

### 2.2. Methods

#### 2.2.1. Three-Step Process of Retentate Treatment

**Figure 1:**
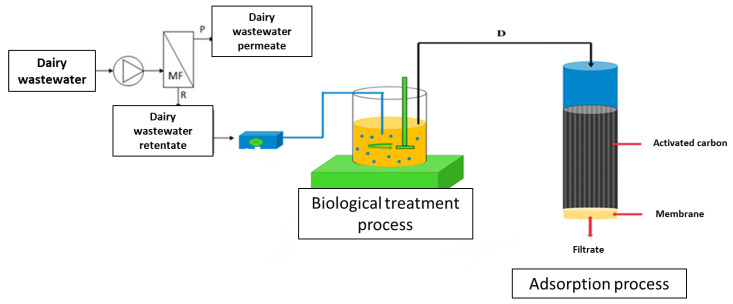
Flow chart of the process of purifying two times concentrated dairy wastewater retentates. MF—Microfiltration module, P—Permeate, R—Retentate, D—Decantate.

As part of the research, dairy wastewater, which is a mixture of post-production wastewater and wastewater from the cleaning of process installations, was subjected to a three-stage treatment process (Figure 1). In the first stage, dairy wastewater was subjected to regeneration processes using a quarter-scale MF installation located at the Łukasiewicz Research Network—Institute of Sustainable Technology (Radom, Poland), presented in previous studies [28]. Prior to charging the MF installation with wastewater, the feed tank, recirculation lines, and 0.2 µm PVDF membrane module had been sanitised and left filled with low-conductivity rinse water (<60 µS/cm). When the 100 dm^3^ batch of dairy wastewater was introduced and recirculation commenced, this residual water mixed with the feed, producing an initial dilution of the circulating retentate. Because the system was operated in batch concentration mode to ~50 dm^3^ final volume, a rigorous component mass balance could not be reconstructed retrospectively from the available grab samples. Nevertheless, 15 cm^3^ grab samples were collected at each stage and analysed in triplicate (see Section 2.2.2), providing reliable comparative concentration data across process streams. 

The MF process was carried out at a constant pressure of 0.5 bar and a feed flow rate of 400 dm^3^/h, utilising a batch system due to changes in the composition of the liquid phase over time and variations in the concentration of components in the feed. The feed tank was filled with 100 dm^3^ of actual dairy wastewater, and the MF process was carried out until a twofold reduction in feed volume was achieved. In this context, the term ‘two–times concentrated retentate’ used throughout the manuscript refers solely to the reduction of the feed volume by half during microfiltration. Specifically, the process began with 100 dm^3^ of wastewater and continued until 50 dm^3^ of retentate remained, which corresponds to a volume-based concentration factor (CF) of 2. Although the manuscript includes concentration data for selected indicators (e.g., COD, TOC), the term “two-times concentrated” is used only in the volumetric sense, and not as an indication of exact doubling of pollutant concentrations. The calculated recovery of the microfiltration process based on volume is 50%.

The efficiency of the MF process was calculated using the following equation:

(2) $$J_{A}=\frac{V}{A\;\times \;t}$$

where:

J_A_ is the permeate flux (at constant temperature and pressure; mL (min × m^2^)^−1^);
V is the volume of filtrate (dm^3^);
A is the effective area of the flat sheet membrane (m^2^);
t is the sampling time (min).

In the second stage, the obtained two times concentrated retentates were subjected to biological treatment. This was carried out using a liquid-phase bioreactor with a capacity of 50.0 dm^3^, equipped with a pressure heating jacket and featuring a set of turbine agitators and sensors for pressure, pH, temperature, oxygen concentration, and liquid level (Figure 1). Activated sludge with an index of 80 cm^3^/g was used in the study. The biological treatment process was carried out for 23.5 h (1 treatment cycle), using the principle of SBR reactors, and divided into four consecutive phases of varying duration: filling (0.5 h), reaction (21 h), sedimentation (1.5 h) and decantation (0.5 h). During the reaction phase, aerobic conditions were maintained by mixing the medium at 250 rpm throughout its duration. After 23.5 h, the obtained decantates were separated from the activated sludge and subjected to physical and chemical analysis.

In the third stage of the analysed treatment process, the decantates after biological treatment were subjected to adsorption in a column containing powdered activated carbon. The sorption process was carried out using a laboratory sorption station consisting of a 20 cm^3^ column secured at the outlet with a non-woven filter (Figure 1). The study used 1 g of PAC, through which 50 cm^3^ of effluent collected after biological treatment was filtered for a contact time of 15 min. The resulting filtrate was then subjected to physicochemical analysis.

#### 2.2.2. Analytics Methods

- Characteristics of the physicochemical properties of process media

The physicochemical properties of actual wastewater, permeate and retentate streams after biological treatment, as well as activated carbon, were characterised. Samples of 15 cm^3^ were taken after each treatment stage, and the following parameters were measured: pH and conductivity (Metler Toledo); turbidity (HACH 2100QiS); chemical oxygen demand (COD, QuickCODLab, Envag); total organic carbon (TOC); total bound nitrogen (TNb, Vario TOC Cube, Elementar); and total phosphorus (TP, HACH LCK 348/LCK 349). Retention at each stage of treatment was then determined based on the obtained parameter values according to the formula:

(3) $$R\;\left[\%\right]=1-\frac{C_{x}}{C_{0}}\times 100\%$$

where:

R—retention of a component at a given stage of purification [%];
C_x_—concentration of individual components at the entrance to each purification stage [mg/dm^3^];
C_0_—concentration of individual components after each purification stage [mg/dm^3^].
All analysed indicators were measured three times.

- Characteristics of powdered activated carbon (PAC)

The evaluation of powdered activated carbon (PAC) properties included characterisation in terms of the following: specific surface area, pore distribution, quality and quantity of functional groups, and structural order. The appropriate research techniques used for these tests included physical sorption (for determining specific surface area and pore distribution; Autosorb Quantachrome, Boynton Beach, FL, USA), Fourier Transform Infrared Spectroscopy (FTIR; Jasco, Tokyo, Japan), and Raman spectroscopy (Jasco, Tokyo, Japan).

## 3. Results and Discussion

### 3.1. First Stage of Purification—MF

In this study, the effectiveness of treating retentates produced after the membrane filtration (MF) process of typical dairy wastewater from washing the production line was evaluated. Table 3 presents the characteristics of the streams after the MF process.

**Figure 2:**
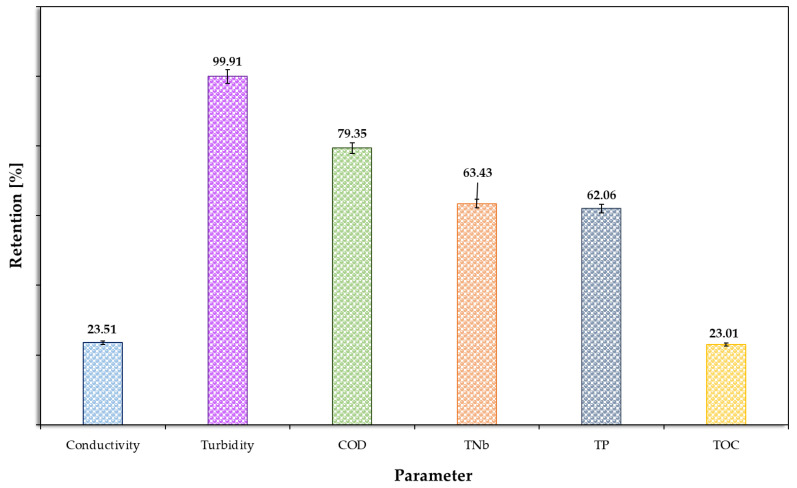
Retention of individual components in permeate.

The MF process was used as a preliminary stage to separate undissolved and colloidal particles. This was performed at a pressure of 0.5 bar and a feed flow rate of 400 dm^3^/h. Permeate flow decreased by 20.6% during the process, compared to an initial value of 20.2 dm^3^/(m^2^ h). The MF process, applied to wastewater from cleaning the dairy product production line, produced two post-process streams: permeate and two times concentrated retentate. Following treatment, slight neutralisation occurred, resulting in a pH level of 7.1. The MF process was found to allow the greatest removal of turbidity (99%), chemical oxygen demand (79%), total nitrogen (63%) and total phosphorus (62%), all of which were concentrated in the retentate, which is presented in Figure 2. The significant reduction in turbidity at a retention level of 99% indicates the removal of mainly proteins and fats, which are present in this type of wastewater. A slight decrease in conductivity and total organic carbon content was observed, with retention at around 23% in both cases. Both biogens (phosphorus and nitrogen) and total organic carbon were concentrated primarily in the retentate. The MF process achieved very high removal of suspended solids and substantial reductions in overall COD when comparing the permeate with the raw feed. In contrast, most dissolved species (conductivity, soluble COD fraction, TNb, TP, TOC) passed through the 0.2 µm membrane; combined with the start-up dilution, this explains why concentrations of these dissolved indicators in the final two times concentrated retentate sample are similar to the raw feed values in Table 2, despite the volume reduction. Accordingly, particulate matter was preferentially retained in the retentate, whereas dissolved components largely appeared in the permeate.

Based on the results of physicochemical analyses, it was concluded that the MF process alone is insufficient to achieve the appropriate levels of specific parameters, by the guidelines of Polish legislation, which is presented in Table 4. It was determined that when using the MF process to treat dairy wastewater from the production line washing, further treatment stages are necessary. The MF process is mainly used as a preliminary treatment stage, replacing bag filtration. Due to the pressure applied and the size of the membrane pores, the MF process primarily retains microorganisms and organic matter, including biogenic compounds [32,33,34], which was also observed in this study (Figure 2). 

According to Polish health regulations [35], process water used in the food industry must meet the standards for drinking water. These standards cover parameters analysed in this study, as well as microbiological contamination, metal content and toxic compounds. In terms of physicochemical properties, the permeates obtained after microfiltration and additional purification using reverse osmosis or advanced oxidation processes could, in the authors’ opinion, be used as process water (Table 4). However, due to the scope of the research, this requires further verification. 

### 3.2. Second Step of the Treatment Process—Biological Treatment

**Figure 3:**
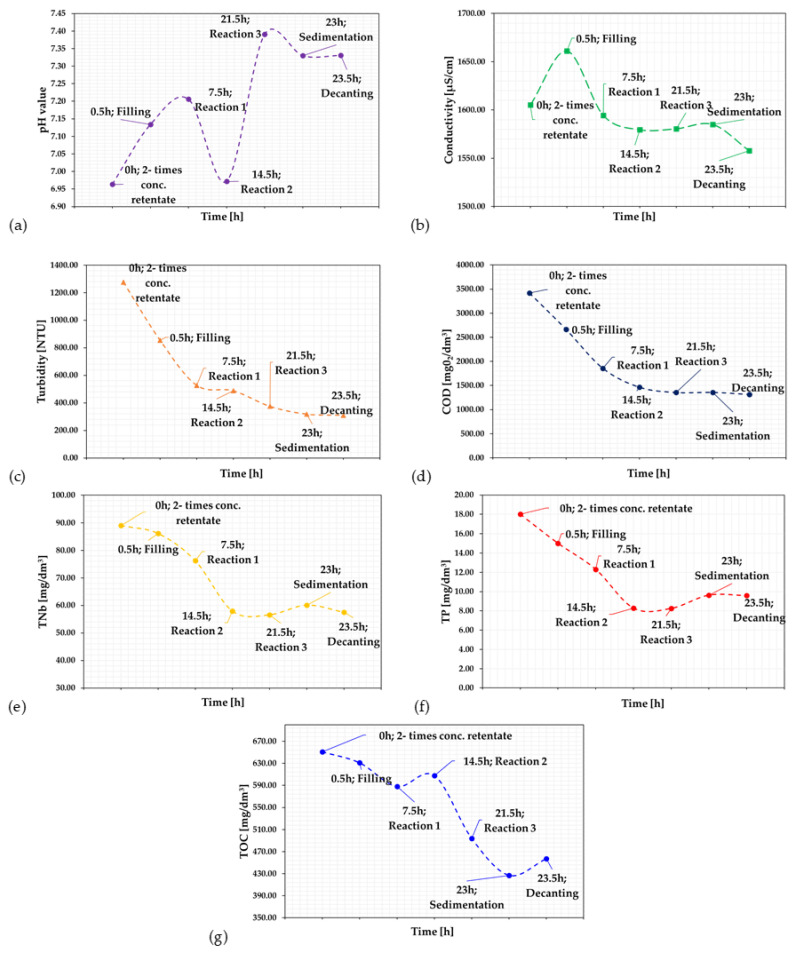
Changes in retention of individual components after 24 h of biological treatment: (**a**) pH; (**b**) conductivity; (**c**) turbidity; (**d**) COD; (**e**) TN_b_; (**f**) TP; (**g**) TOC.

In the next stage, the two times concentrated retentate after MF was subjected to biological treatment using activated sludge (MLSS of 2500 mg/dm^3^). This process lasted 24 h and was divided into several stages. After each stage, samples were taken for testing of physicochemical parameters, after waiting approximately 10 min. For the aerobic reaction phase, which lasted 21 h, samples for physicochemical analysis were taken every 7 h to verify the retention of individual components (designation of individual sampling as reaction 1, reaction 2 and reaction 3). Figure 3 shows the retention of the analysed parameters.

The study observed that the highest retention was achieved for the removal of suspended solids during the entire purification process, with over 75% being removed in the decantation phase (Figure 3). The pH value increased with treatment duration (Figure 3a). The highest value for this parameter was achieved at the end of the reaction phase. The slight decrease in pH during decantation was probably caused by the settling of organic matter residues, whose particles could have affected the pH value of the samples analysed during the reaction phase. The initial increase in conductivity was attributed to the mixing of dairy wastewater with activated sludge, leading to the release of ionic compounds into the liquid phase. As the biological treatment progressed, a gradual decline in conductivity was observed, with the most significant reduction occurring within the first 7 h. During the subsequent hours, the conductivity remained relatively stable. A slight decrease was also recorded during the decantation phase, which may be explained by the residual influence of suspended biomass affecting the measurement. The overall reduction in conductivity is most likely associated with the assimilation of ionic nutrient species by microbial biomass, subsequently utilised for cellular growth. The use of activated sludge treatment allowed for a reduction of turbidity by approximately 75% compared to the initial stream, i.e., twice the concentration of retentate [36].

After 23.5 h of activated sludge treatment under experimental conditions, reductions of nearly 62% in COD and 30% in TOC were achieved. The most significant reduction in chemical oxygen demand (COD) occurred within the first 14.5 h of the biological treatment process, amounting to approximately 50% (Figure 3d). In the following stages, the rate of COD removal decreased, which may be attributed to the exhaustion of readily biodegradable organic compounds [37]. Continuous degradation of organic matter by the activated sludge was also confirmed by the consistent decrease in the total organic carbon (TOC) concentration (Figure 3g). The TOC value dropped during the initial 7.5 h and then exhibited a slight increase at 14.5 h. This temporary rise is likely due to the hydrolysis of complex organic compounds, such as lipids and proteins, leading to the release of soluble forms of organic carbon [8]. A slight decrease in pH observed at 14.5 h of the biological treatment process may further support this hypothesis. Following this point, TOC values continued to decline, which can be explained by the assimilation and metabolic activity of the microbial community acting on the simpler, hydrolysed organic carbon fractions. 

Biological treatment is one of the most common methods of treating municipal and industrial wastewater, which mainly contains biodegradable organic matter. Microorganisms consume the compounds in wastewater as a source of food and for their metabolic processes. The treatment principle is the same as that of the natural self-purification of water reservoirs. The difference lies in creating optimal conditions for the process, such as the presence of oxygen, nutrients, mechanical mixing, and maintaining the correct temperature and pH. These conditions increase the speed and efficiency of the process [3]. Dairy wastewater, particularly from cleaning production lines, is a challenging category characterised by high fat and protein content and high COD levels ranging from 500 mg/dm^3^ to 102,100 mg/dm^3^ [38]. The reduction in COD, TOC and turbidity achieved within a given time and with such a pollutant load allows us to conclude that satisfactory treatment results can be achieved with activated sludge (see Table 5). Conducting another treatment cycle with a supply of fresh, two times concentrated retentate would likely allow for greater retention of these pollutants, but this requires verification in further experiments.

The 24-h activated sludge treatment process removed over 46% of phosphorus and over 35% of nitrogen (see Table 4). Removing nitrogen and phosphorus compounds from wastewater is important because high levels can contribute to the eutrophication of watercourses and pre-treated wastewater reservoirs, which are to be further treated at a later stage. As illustrated in Figure 3e,f, a consistent decrease in TN_b_ and TP concentrations was observed throughout the biological treatment process. The most pronounced reduction for both parameters occurred within the first 14.5 h of the reaction phase. Subsequently, their concentrations remained relatively stable. The removal of TN_b_ and TP was most likely driven by microbial assimilation by activated sludge. The stabilisation of TN_b_ and TP reduction may be attributed to suboptimal oxygen conditions during the reaction period. Mechanical mixing alone may have enabled limited oxygen transfer from the surface, resulting in insufficient aeration. Consequently, this could have hindered complete denitrification while allowing only partial nitrification. In the case of phosphorus, in the absence of a defined anaerobic zone, the removal likely proceeded primarily via adsorption onto activated sludge flocs. As demonstrated in other studies, treating dairy wastewater with traditional activated sludge and oxygen primarily reduces the biological load, as well as the nitrogen, phosphorus, protein and fat content [3,39,40]. Analysis of changes occurring during a 24-h treatment cycle using activated sludge indicates high microbial activity, particularly during the reaction phase, where aerobic conditions were employed (Table 4).

### 3.3. Third Step of the Treatment Process—Adsorption of PAC

**Figure 4:**
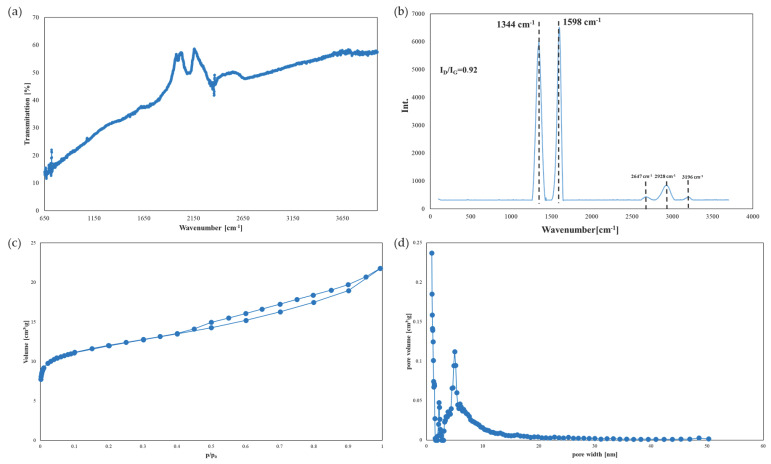
FTIR spectrum (**a**) and Raman spectrum (**b**) for PAC-activated carbon; N2 adsorption-desorption isotherm at 77K (**c**) and pore distribution (**d**).

The obtained decantations after the biological treatment were subjected to adsorption in laboratory columns containing 1 g of PAC carbon (concentrated of PAC adsorbent 20 g/dm^3^). The structural properties of the commercial activated carbon were characterised to determine its suitability for purifying this type of medium. Figure 4 shows the Raman and FTIR spectra of the PAC carbon. Within the analysed wavelength range, no characteristic bands originating from acidic or basic groups, which may be present on the surface of sorbent materials, were detected (Figure 4a).

Raman spectrum analysis of PAC carbon confirms the presence of the two characteristic D and G bands of carbon materials at 1344 and 1598 cm^−1^, respectively (Figure 4b). The material is characterised by a high degree of disorder, as evidenced by the D/G band intensity ratio of 0.92. Additionally, bands appearing in the 2647–3196 cm^−1^ range indicate this carbon material’s highly amorphous structure [41]. Analysis of the N_2_ adsorption-desorption isotherm indicates a developed specific surface area. The specific surface area of the PAC carbon was determined using the Brunauer–Emmett–Teller (BET) method. In contrast, the pore size distribution and micropore capacity were determined using the density functional theory (DFT) and t-plot methods, respectively. PAC carbon has a specific surface area of 969 m^2^/g, with micropores averaging 0.926 nm in size, with a capacity of 0.268 cm^3^/g. Micropores account for nearly 70% of the surface area of PAC. This structure indicates that pollutant sorption will be physical, mainly resulting from van der Waals interactions [42].

**Figure 5:**
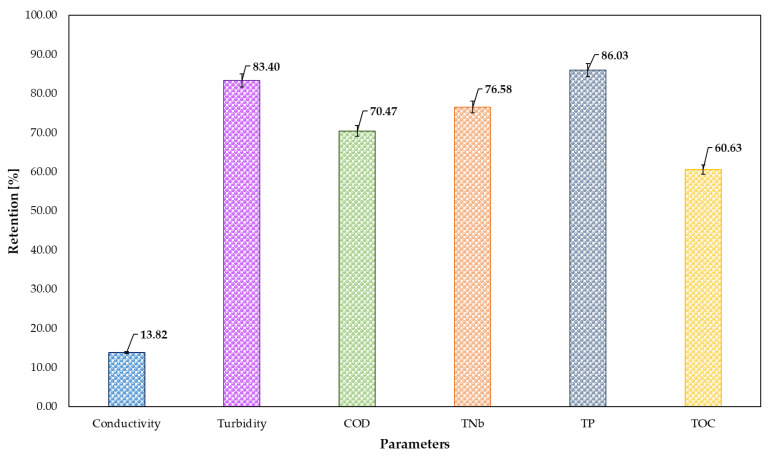
Retention of individual components after the sorption process.

Next, the decantate obtained after the biological treatment process was subjected to sorption on a bed containing 1 g of activated carbon. The sorption process was carried out for 30 min, after which the retention level of the analysed parameters was determined. Additional treatment of the decantate after biological treatment significantly reduced the content of almost all components, except for conductivity, which showed a 14% retention, as shown in Figure 5. The highest percentages of retention were achieved for TP and suspended solids, at 86% and 83%, respectively. Adsorption of nitrogen compounds was close to 77%, while COD was around 70%.

The adsorption process can be divided into three main stages: external diffusion, internal diffusion and a surface reaction involving the attachment of the adsorbate to the adsorbent [43]. A study by Streit et al. [44] has demonstrated that the size of the adsorbate molecule significantly affects the binding of pollutants by activated carbon. Larger molecules have lower adsorption process dynamics due to their difficulty in penetrating pores or occupying adsorption sites, which can reduce their adsorption capacity compared to smaller molecules [44]. Furthermore, the smaller the activated carbon grains and the higher the proportion of mesopores, the more effectively organic substances are removed [45]. The PAC used in this study has a micro-mesoporous structure, in which mesopores constitute approximately 30% of the surface area (see Figure 4). This structure enables the retention of both smaller molecules, such as nitrogen and phosphorus, and larger organic substances, as demonstrated by the retention of suspended solids and chemical oxygen demand (COD). Other studies have verified that activated carbons can retain various organic substances, with retention levels ranging from 60 to 99% [46,47,48]. In this study, higher retention was achieved for smaller molecules, as confirmed by the TNb and TP results. Similar results were obtained by Wang et al., who analysed the ability of various carbon materials to absorb nitrogen and phosphorus from water. They demonstrated that phosphorus adsorption is more efficient than nitrogen adsorption [49]. This depends on various factors, including the pH of the solution, the process temperature, and the presence of other cations and anions, which can significantly affect the adsorption of these components onto activated carbon.

## 4. Conclusions

The aim of the present study was to evaluate the feasibility of applying a combined activated sludge–adsorption system for the treatment of two times concentrated MF retentates derived from mixed dairy wastewater. The MF process is commonly employed as a preliminary treatment step, generating two separate streams: permeate and retentate. Both streams were characterised in terms of basic physicochemical parameters, including turbidity, conductivity, COD, TOC, TP and TNb. The two times concentrated retentate was selected for further treatment. The first stage of the treatment process involved biological purification in a sequencing batch reactor (SBR). After 23.5 h of operation, the final decantate showed substantial reductions in turbidity (75.2%), COD (61.6%), TOC (29.8%), TP (46.8%) and TNb (35.4%). The second stage involved adsorption onto powdered activated carbon (PAC) over a 30-minute contact time. Analysis of parameter retention confirmed that the selected PAC effectively adsorbed suspended solids (83.4%), which contributed significantly to the observed reductions in COD (70.5%) and TOC (60.6%). In addition, notable retention of nitrogen (76.8%) and phosphorus compounds (86.0%) was observed relative to the decantate from the biological stage. The combined two-step treatment of the concentrated retentates resulted in retention efficiencies ranging from 24.63% to 97.03%, with the most pronounced reductions achieved for suspended solids (97.03%), total phosphorus (87%) and COD (84%). Despite the high removal efficiencies, the final effluents did not meet the discharge requirements defined by Polish environmental regulations. Therefore, future research will focus on optimising and improving the treatment process to allow safe discharge of treated retentates into the environment.

## Figures and Tables

**Table 1:** Characteristic of raw dairy wastewater.

Parameter	Unit	Raw Sewage
pH	-	6.76 ± 0.05
Conductivity	µS/cm	1785.10 ± 18.42
Turbidity	NTU	1740.87 ± 21.12
COD	mg/dm^3^	3616.00 ± 26.4
TN_b_	mg/dm^3^	83.40 ± 4.1
TP	mg/dm^3^	16.23 ± 0.31
TOC	mg/dm^3^	644.70 ± 0.08

**Table 2:** Characteristic of the used membrane for microfiltration.

Parameters	MF Membrane
Manufacturer	TriSept Membranes
Model	TM10-QXF
Polymer	PVDF
Area, m^2^	5.8
Pore size, µm	0.2
Molecular weight cut-off, kDa	-
Max. pressure, bar	21
Max. temperature, °C	45

**Table 3:** Physicochemical properties of streams after the MF process—comparison with Polish requirements for wastewater discharge into waters and soil.

Parameter	Unit	Permeate	2 Times Conc. Retentate	Required Levels [31]
pH	-	7.10 ± 0.13	6.96 ± 0.07	6.5–9.0
Conductivity	µS/cm	1356.40 ± 34.58	1602.80 ± 23.61	-
Turbidity	NTU	1.47 ± 0.06	1259.00 ± 39.88	-
COD	mg/dm^3^	758.67 ± 21.55	3454.00 ± 34.01	125
TN_b_	mg/dm^3^	31.67 ± 3.21	86.00 ± 3.07	30
TP	mg/dm^3^	6.04 ± 0.21	17.74.00 ± 0.44	2
TOC	mg/dm^3^	493.07 ± 8.46	648.07 ± 4.99	30

**Table 4:** Comparison of the physicochemical properties of permeate with the levels required by Polish law in the context of meeting the requirements for water for reuse.

Parameter	Unit	Permeate	Required Levels [35]
pH	-	7.10 ± 0.13	6.5–9.5
Conductivity	µS/cm	1356.40 ± 34.58	2.500
Turbidity	NTU	1.47 ± 0.06	Up to 1.0
COD	mg/dm^3^	758.67 ± 21.55	-
TN_b_	mg/dm^3^	31.67 ± 3.21	-
P_og_.	mg/dm^3^	6.04 ± 0.21	-
TOC	mg/dm^3^	493.07 ± 8.46	No abnormal changes

**Table 5:** Changes in the levels of the analysed parameters in individual phases of the biological treatment process.

Parameter	Unit	2-Times Conc. Retentate	Filling	Reaction 3	Sedimentation	Decanting
pH	-	6.96 ± 0.07	7.15 ± 0.07	7.39 ± 0.02	7.30 ± 0.03	7.30 ± 0.02
Conductivity	µS/cm	1602.80 ± 23.61	1621.03 ± 34.76	1573.83 ± 28.10	1576.33 ± 18.58	1561.27 ± 39.11
Turbidity	NTU	1259.00 ± 39.88	847.33 ± 29.57	365.63 ± 10.65	315.93 ± 11.76	311.60 ± 8.03
COD	mg/dm^3^	3454.00 ± 34.01	2651.33 ± 34.78	1348.33 ± 21.77	1343.67 ± 12.58	1319.67 ± 24.01
TN_b_	mg/dm^3^	86.00 ± 3.07	85.77 ± 0.42	55.77 ± 1.27	59.43 ± 0.91	56.50 ± 0.95
TP.	mg/dm^3^	17.74.00 ± 0.44	14.96 ± 0.49	8.21 ± 0.13	9.53 ± 0.11	9.40 ± 0.17
TOC	mg/dm^3^	648.07 ± 4.99	630.57 ± 1.02	494.19 ± 0.41	427.33 ± 0.59	456.65 ± 0.65

## Data Availability

The original contributions presented in the study are included in the article, further inquiries can be directed to the corresponding author.
